# Phenotypic Response of *Wolbachia pipientis* in a Cell-Free Medium

**DOI:** 10.3390/microorganisms8071060

**Published:** 2020-07-16

**Authors:** Alyssa M. Krafsur, Arnab Ghosh, Corey L. Brelsfoard

**Affiliations:** Department of Biological Sciences, Texas Tech University, 2901 Main St., Lubbock, TX 79409, USA; alyssa.krafsur@ttu.edu (A.M.K.); Arnab.Ghosh@ttu.edu (A.G.)

**Keywords:** axenic media, phenotypic microarray, Aa23, *Wolbachia*

## Abstract

*Wolbachia*, an obligate intracellular bacterium estimated to infect millions of arthropod species worldwide, is currently being utilized in novel control strategies to limit the transmission of Dengue and Zika viruses. A limitation for *Wolbachia*-based control approaches is the difficulty of transferring *Wolbachia* to novel hosts and the lack of tools for the genetic transformation of *Wolbachia* due to the inability to culture *Wolbachia* outside the insect host cell in an axenic media. Here, we applied extracellular *Wolbachia* to phenotypic microarrays to measure the metabolic response of *Wolbachia* in media formulations with different pH levels and supplementation with Casamino acids. Results suggested a pH of 6.5–6.8 and showed that the supplementation of 1 mg/mL casamino acids increased the survival and longevity of *Wolbachia* in an axenic medium. In addition, phenotypic microarrays are a useful tool to measure the phenotypic response of *Wolbachia* under different media conditions, as well as determine specific components that may be required for an axenic medium. This study is an initial step toward the development of a potential *Wolbachia* axenic culture system.

## 1. Introduction

*Wolbachia pipientis* is an obligate intracellular maternally inherited bacterium found in >55% of insects, as well as filarial nematodes and terrestrial crustaceans [1,2]. In insects, *Wolbachia* alters host reproduction with several phenotypes, including feminization, parthenogenesis, male killing, and cytoplasmic incompatibility (CI) [3]. The discovery of these reproductive alterations, particularly CI, has caused *Wolbachia* to be a bacterium of interest for vector control. CI is used to drive desired phenotypes, such as refractoriness to disease transmission, with the goal of replacing natural infected populations [4,5,6,7,8]. In addition, *Wolbachia*-induced CI is also currently used as part of an incompatible insect technique (IIT) [9] strategy, where males harboring incompatible infections are released with the goal of suppressing natural populations by incompatible mating. An unexplored *Wolbachia* based approach centers around paratransgenesis, or the process of decreasing vector competence through genetically modifying an organism’s symbionts. Due to the maternal transmission of *Wolbachia* and the reproductive advantage afforded by *Wolbachia*, it may be possible to use paratransgenesis to introduce a transgene into a population, in turn producing an antipathogenic molecule to block the transmission of vectored pathogens [8,10,11,12]. However, the genetic transformation of *Wolbachia* has not been achieved, due to the difficulty of delivering genetic constructs into an intracellular symbiont.

Through the observed horizontal transmission events, it is reasoned that *Wolbachia* must have spent a portion of their life cycle outside of their host. Empirical studies subsequently demonstrated *Wolbachia* can live outside of its host and retain viability for up to seven days [13]. Studies in vivo demonstrated the presence of *Wolbachia* in the hemolymph of both larvae and adults of *Drosophila* and mosquitoes, which provides further evidence for the idea that *Wolbachia* can survive extracellularly [14,15,16,17]. It would be beneficial from a research perspective for *Wolbachia* to replicate outside of the host cell, so the bacterium can be better studied and made easily available for transfection strategies. However, cellular replication has never been observed outside of its host cells, suggesting metabolic limitations or replication factors are not available or produced outside of the insect cells to support *Wolbachia* replication.

*Wolbachia* has a reduced genome similar to other intracellular symbionts and pathogens, which is consistent with the hypothesis that *Wolbachia* relies on its host cell machinery for energy sources and other metabolic factors [18,19,20,21]. Genomic studies have suggested the metabolic properties of *Wolbachia* are similar to *Rickettsia* bacteria, meaning *Wolbachia* may have limited carbohydrate metabolism, participate in lipid synthesis, and depend on amino acid transport [18,19,20,21]. Recent studies have also suggested that *Wolbachia* titers in host tissue are dependent upon ubiquitin and endoplasmic reticulum associated protein degradation pathways (ERAD) to supply sufficient amino acids for growth and reproduction [22]. The results of these studies also suggest the absence of glycolytic and gluconeogenic enzymes, the reduced pathways for amino acid biosynthesis, and the presence of transporters for proline, aspartate/glutamate, and alanine, which again suggests a reliance on host amino acids as a major energy source [18,23]. 

The creation of a cell-free media to support intracellular endosymbiont growth has been accomplished before. Studies of the predicted metabolic capacities of bacteria from the genera *Coxiella, Chlamydia,* and *Rickettsia* and other obligate symbionts suggest a common nutritional deficiency of amino acids that is compensated for by the activities of amino acid and peptide permeates that scavenge amino acids from their hosts [24]. Particularly, the Q fever bacterium *Coxiella burnetii* was systematically evaluated for conditions to support extracellular growth [25], leading to the development of a complex nutrient medium that supported replication and growth of the bacteria. Another factor to consider when constructing a cell-free media is the pH required for growth. The pH level for insect cell culture growth is well defined depending on the insect cell type, but intracellular pH levels can vary depending upon the media type and media pH. Insect cell lines have been shown to have the highest growth rates and maximum densities at a pH of 6.2–6.8 [26]. In the presence of an acidic media, insect cells have been shown to have a capacity to buffer pH inside of cells and maintain a neutral pH level of 7.0 [27]. Previous attempts to isolate *Wolbachia* and examine replication were performed in Schneider’s media at a pH of 6.2–6.5, which may be optimal for insect cells that can buffer the pH level, but not extracellular *Wolbachia*, which may require pH levels in an axenic media to be similar to intracellular levels of 6.5–7.0. Axenic growth of *C. burnetii* has fueled important new areas of research to aid in understanding disease transmission, as well as a new set of genetic tools for functional genetic studies of the pathogen [28,29,30,31,32,33]. Similar to *C. burnetii,* a *Wolbachia* axenic culture and associated toolset have the potential to open similarly broad vistas of research.

Besides what is predicted from genomic data, little knowledge of host factors and metabolic requirements for *Wolbachia* survival and replication is available. Using a similar approach as that which was successful for the development of other axenic intracellular bacteria cultures [34,35,36,37,38,39], we attempted to determine general host factors and metabolic requirements involved in *Wolbachia* survival outside of its host cell. Here, we used a phenotypic microarray approach to examine the response of *Wolbachia* in an axenic culture. Specifically, we altered the pH and casamino acid concentration in an axenic medium and measured the metabolic response of extracellular *Wolbachia* using phenotypic microarrays. Results will be useful for the future testing and identification of the conditions required for *Wolbachia* survival and extracellular growth in an axenic media.

## 2. Materials and Methods

### 2.1. Cell Culture

The *w*AlbB infected Aa23 cell line used in experiments was obtained from Dr. Zhiyong Xi [40]. Cells were cultured in 25 cm^2^ or 75 cm^2^ cell culture flasks (Techno Plastic Products, Trasadingen, Switzerland) in Schneider’s insect medium (SM) (MilliporeSigma, St. Louis, MO, USA) (Table S1) supplemented with 10% fetal bovine serum (FBS). All cell cultures were incubated at 28 °C with a CO_2_ concentration of ~0.2% (ambient atmospheric concentration). Cells were passaged at a 1:4 cell to media ratio every seven days. Aposymbiotic Aa23-T cells were generated by treating Aa23 cells with 3 mg/mL tetracycline for three passages, and subsequently referred to as Aa23-T. 

### 2.2. Trypan Blue Staining and Hemocytometer Aa23 Cell Counts

To determine cell counts prior to *Wolbachia* isolations, 200 µL of infected Aa23 cells were subjected to a centrifugation step at 200× *g* for 10 min. The pellet was then resuspended in 200 µL of 1× phosphate-buffered saline (PBS) solution. The cellular resuspension was then diluted in a 1:1 ratio with 0.4% Trypan Blue stain (Gibco, Carlsbad, CA, USA). Approximately 200 µL of isolated *Wolbachia* was centrifuged for five minutes at 16,000× *g* and the pellet was also resuspended in 1× PBS solution and diluted with Trypan blue in an identical 1:1 ratio. After five minutes, 10 µL of the two suspensions were placed in a hemocytometer and cells counted using a DMIL LED inverted microscope (Leica biosystems, Wetzlar, Germany) at 20× magnification. The hemocytometer grid images were captured using a 3 MP USB 2.0 Color CMOST Camera and software (v3.2.0000) (Amscope, Irvine, CA, USA). 

### 2.3. Confirmation of Wolbachia Infection Status Using PCR

A DNeasy Blood and Tissue kit (Qiagen, Hilden, Germany) was used to extract DNA from cell lines and isolated extracellular *Wolbachia* following manufacturer’s instructions. *Wolbachia*-specific primers were used to confirm the presence of *Wolbachia* in both intracellular and extracellular conditions [41,42,43,44,45]. Polymerase chain reaction (PCR) was also used to check for contamination of *Acinetobacter* spp., *Cardinium* spp., and *Asaia* spp. All primer sets to confirm *Wolbachia* infections and other bacterial contamination are listed in Supplementary Table S2. For all reactions, 1 µL of isolated DNA was amplified in 25 mM of KCL, 25 mM of Tris-HCL (pH 9.0), 20 mM of (NH_4_)_2_SO_4_, and 0.025% Triton X-100, 0.25 mM of MgCl_2_, 0.25 mM of dNTPs, 0.5 mM of primers, and 1 µL of Taq DNA polymerase in a total volume of 25 µL. The PCR amplification protocol was 10 min at 95 °C, 35 cycles of 30 s at 95 °C, 30 s at 55 °C, and 1 min at 72 °C, followed by a 10-min extension step at 72 °C. The PCR reaction was run on a T100 Thermocycler (Bio-Rad laboratories, Hercules, CA, USA). All reactions products were visualized on a 1.52–% agarose gel, stained with GelRed (Biotium, Fremont, CA, USA), and visualized under ultraviolet illumination.

### 2.4. Fluorescent In-Situ Hybridization

Fluorescent in-situ hybridization (FISH) was performed on the Aa23 and Aa23-T cell lines to confirm the presence and absence of *Wolbachia*, respectively. Cells were grown to 90% confluency at 28 °C and 300 µL of the cells were added to four wells of an eight-well Nunc Lab-Tek™ Chamber slide system (ThermoFisher Scientific, Waltham, MA, USA). At 24 h post-extraction, isolated *Wolbachia* cells were added to the two of the other wells and Aa23-T cells were added to the two remaining wells. The cells were incubated in the chambered wells overnight for 15 h at 28 °C. Cells were taken out the next day and fixed in 4% formaldehyde (in 1× PBS) for 40 min at room temperature (RT). This was followed by two washes with 1× PBS. Next, the cells were prehybridized for ~ 2 h at RT. The prehybridization buffer consisted of 50% deionized formamide, 20% 20× sodium saline citrate (SSC) solution, 1% 50× Denhardt’s Reagent, 10% 1 mol of dithriothreitol (DTT), 0.25 of mg/mL t-RNA, and 0.25 mg/mL of poly(A). The prehybridization step was followed with overnight hybridization (~ 18 h) at 37 °C with gentle shaking. The prehybridization buffer was supplemented with 200 mg/mL of dextran sulfate, 250 mg/liter of salmon sperm DNA, and *Wolbachia*-specific probes (5′-/56-FAM/AATCCGGCCGARCCGACCC-3′); 5′-/56-FAM/CTTCTGTGAGTACCGTCATTATC-3′) [13]. After hybridization, the cells were washed with denatured SSC solution in the following order: Wash buffer 1 (1× SSC augmented with 10 mmol/liter DTT) at RT with gentle shaking, wash buffer 1 at 55 °C with gentle shaking, and two washes at 55 °C with buffer 2 (0.5× SSC augmented with 10 mmol/liter DTT) with gentle shaking. Following the wash steps, cells were stained with DAPI at room temperature for 5 min followed by three washes with 1× PBS for 5 min each. The cells were then observed under a BX-41 epifluorescence microscope (Nikon, Tokyo, Japan) with appropriate filters at 60x magnification. Images were captured and processed with the help of QCapture™ software (v2.0.12) (Teledyne Qimaging, Surry, British Columbia, Canada). 

### 2.5. Isolation of Extracellular Wolbachia

**Figure 1:**
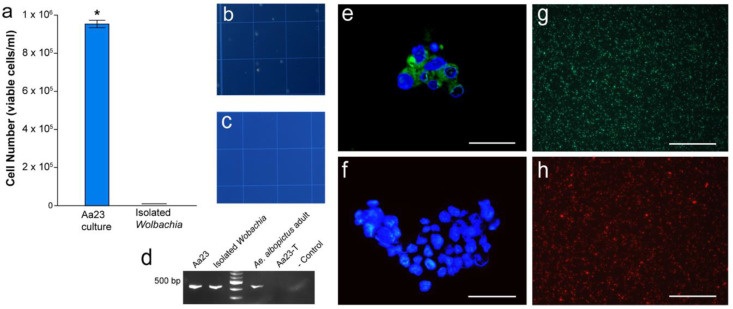
(**a**) Aa23 cell counts prior to *Wolbachia* isolation procedures. * above the bars represents a significant difference (t-test, *p* < 0.05), (**b**) pre- and (**c**) post-*Wolbachia* isolation using Trypan Blue stain, (**d**) Polymerase chain reaction (PCR) confirmation of *Wolbachia* infections in Aa23 cells and isolations, (**e**) Fluorescent in-situ hybridization (FISH) staining of Aa23 cells confirming *Wolbachia* infection, (**f**) FISH staining of Aa23-T aposymbiotic cells showing the absence of a *Wolbachia* infection, and (**g**) Baclight LIVE/DEAD™ staining of *Wolbachia* immediately after isolation procedures, and (**h**) Baclight LIVE/DEAD™ staining of heat killed *Wolbachia*. White scale bars represent 50 µM.

To isolate the *w*AlbB infection, a mean of 9.59 × 10^5^ ± 1.97 × 10^4^ cells was normalized during the log growth phase and used for each isolation procedure (Figure 1a). Extracellular *Wolbachia* was isolated using a modified procedure previously described [13]. Before the final centrifugation step, 200 µL of a 250-mM sucrose solution was added to each milliliter of isolated *Wolbachia* solution. The final centrifugation step was performed at 17,000× *g* for 10 min. The resulting pellet contained extracellular *Wolbachia* and after discarding the supernatant, the pellet was resuspended in SM + 10% fetal bovine solution (FBS) and passed through a 2.7-µM filter. To examine for potential environmental contamination, 20 µL of the isolation solution was streaked on LB agar and incubated for 48 h at 28 °C. 

### 2.6. Epifluorescence Microscopy

To confirm the isolation of extracellular *Wolbachia,* a Baclight LIVE/DEAD™ bacterial viability kit was used to visualize the bacterium (ThermoFisher Scientific, Waltham, MA, USA). SYTO9 and propidium iodide (PI) were used to differentiate the live and dead bacterial cells following manufacturer’s instructions. Briefly, 100 µL of each of SYTO9 and PI were pre-mixed to prepare the stain mix and 10 µL of the extracellular *Wolbachia* was added to the stain mix in a 1:1 ratio. This mixture was incubated in the dark for 10 min. The bacteria-stain mix (20 µL) was then placed on a glass slide, covered with a coverslip, and viewed using a Nikon BX41 inverted microscope at 100× magnification. To confirm staining efficacy, extracellular *Wolbachia* were heat-killed at 95 °C for 15 min and subsequently stained. Images were processed with QCapture™ software (v2.0.12) (Teledyne Qimaging, Surry, British Columbia, Canada).

### 2.7. Phenotypic Microarray Assays 

Two categories of phenotypic microarray (PM) plates, PM1 carbon source plates and PM3B nitrogen source plates (Biolog, Hayward, CA, USA), were used to assess the metabolic activity of extracellular *Wolbachia* [46]. Biological replicates of each plate type and treatment were completed in duplicate or triplicate. For treatment plates, 100 µL of isolated *Wolbachia* in post-isolation media (SM supplemented with 10% FBS with either an adjusted pH or the addition of casamino acids) was added to each well with 110 µL of Redox Mix G (Biolog, Hayward, CA, USA). Control plates for the normalization of background absorbance consisted of post-isolation media at a pH of 6.8 for both PM1 and PM3B microarray plate assays. PM1 and PM3B microarray plates were incubated at 28 °C with a CO_2_ concentration of ~0.2% for four days. To examine for an effect of pH on the survival of the extracellular *Wolbachia* in an axenic medium, the unbuffered pH of the post-isolation media (~ 6.8) was adjusted to 6.5, 7, and 7.5, respectively. The resulting media was filter sterilized using a 0.2-µm filter before being used for the isolation of *Wolbachia*. To examine for an effect of casamino acids on extracellular *Wolbachia*, the post-isolation media was supplemented with 1 mg/mL, 2 mg/mL, and 4 mg/mL of casamino acids (VWR chemicals, Solon, OH, USA) and filter sterilized. The absorbance measured for each well on the PM plates was at an O.D. of 600 in a Biotek Synergy H1 Microplate Reader (Biotek, Winooski, VT, USA). Absorbance measurements were analyzed using a Biotek Gen 5 Microplate Reader and imager software v.3.02 (Biotek, Winooski, VT, USA). All absorbance measurements were taken every 24 h over a period of four days. To normalize absorbance values and to account for background noise from the isolation medium, absorbance values from control plates were subtracted from the treatment plate absorbance values for each plate type and replicate.

### 2.8. Quantitative Real-Time PCR Analysis

Extracellular *Wolbachia* was quantified using quantitative polymerase chain reaction (qPCR). DNA was isolated using a Qiagen DNeasy kit (Qiagen, Hilden, Germany) following the manufacturer’s instructions. qPCR was performed using PowerUp™ SYBR™ Green Master Mix (Applied Biosystems, Foster City, CA, USA) following manufacturer instructions with an ABI 7300 real-time qPCR system (Applied Biosystems, Foster City, CA, USA). Primers used for reactions are listed in Table S2. *Wolbachia* absolute cell counts were quantified by performing a serial dilution of an Aa23 cell culture sample and the generation of a standard curve. Three biological replicate samples were amplified in triplicate and the average C_t_ values were used to quantify *Wolbachia*.

### 2.9. Wolbachia Cell Counts

To confirm the isolation of extracellular *Wolbachia* a Baclight LIVE/DEAD™ bacterial viability kit (ThermoFisher Scientific, Waltham, MA, USA) was used to differentiate live and dead bacterial cells following manufacturer’s instructions. Ten µL of the stained solution was placed on a slide with a cover slip and imaged at 100× using a Nikon BX41 inverted microscope with epifluorescence. Viable *Wolbachia* cells stained green (SYTO9) and dead cells stained red (PI). Viable cells were counted using three representative images using Image-J.

### 2.10. Statistical Analyses 

All statistical analyses were performed using JMP software (SAS, Cary, NC, USA). Statistical difference of *Wolbachia* counts using qPCR at each timepoint were determined using t-tests, with a significance level of *p* < 0.05. Heatmaps were generated using mean absorbance values from replicate phenotypic microarray assays. All data from phenotypic microarrays for the different treatment levels of casamino acids and pH were combined into a single data matrix. Differences between phenotypic microarray treatment groups within each time point were determined using a one-way analysis of variance (ANOVA). Differences in cell counts were determined using Kruskall–Wallis and pairwise Mann–Whitney tests. Difference between treatment levels were considered to be significant when the *p*-value was < 0.05. 

## 3. Results

### 3.1. Confirmation of Wolbachia Infections and Isolation of Wolbachia

To determine that only *Wolbachia* was in the isolate and there were no residual cells, Aa23 cells isolates were stained with trypan blue. An analysis of the ratio of white (living) to blue (dead) Aa23 cells from three independent observations showed no Aa23 cells were observed in the *Wolbachia* isolate (Figure 1b,c). *Wolbachia* infections were confirmed in Aa23 cells and isolates using PCR and FISH (Figure 1d–f). Live *Wolbachia* was observed immediately after isolation using an SYTO9 stain as shown by green cells (Figure 1g). In addition, a heat-killed isolated *Wolbachia* sample showed only PI red-stained cells (Figure 1h). *Aedes albopictus* is known to harbor other bacterial symbionts, including *Cardinium* spp., *Asaia* spp., and *Acinetobacter* spp. [42,47], which could confound metabolic assays. Results from PCR assays suggest that isolates contained no *Asaia* spp., *Cardinium* spp., or *Acinetobacter* DNA (Table S3). Streaking of the *Wolbachia* isolate on LB plates also showed no evidence of contamination resulting from the isolation procedure. Plates remained colony-free after the 48-h incubation period.

### 3.2. Phenotypic Microarray Assays 

**Figure 2:**
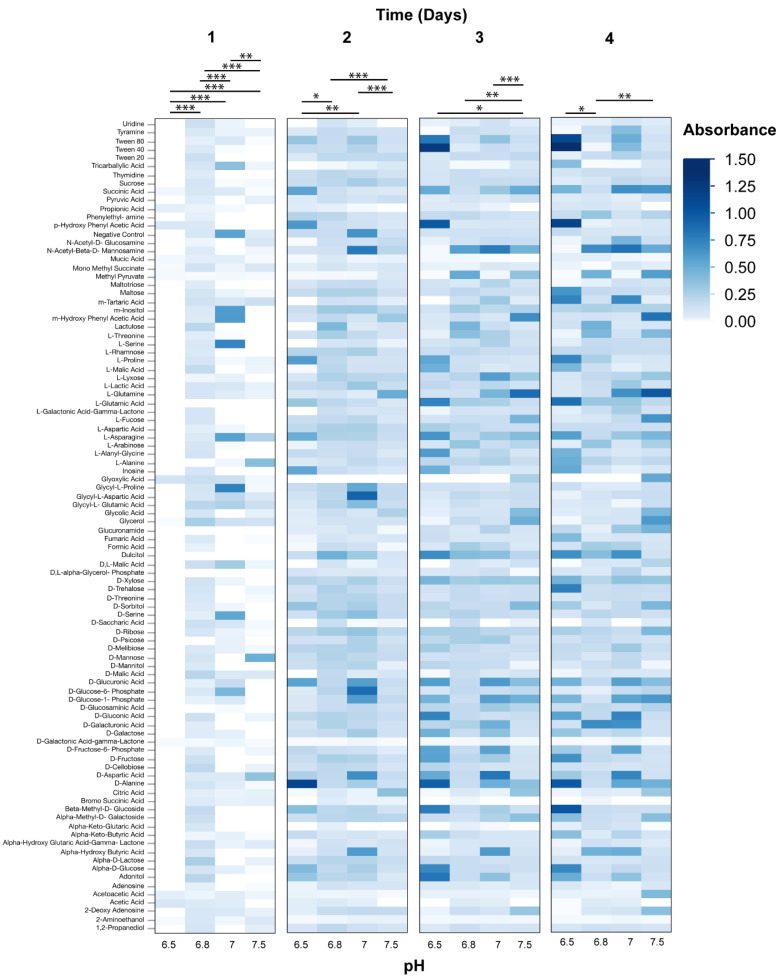
Heat map of PM1 phenotypic microarray data from *Wolbachia* isolated and maintained in Schneider’s medium supplemented with 10% FBS at different pH levels (*n* = 2 for each treatment). All data were normalized by subtracting absorbance values of control plates containing Schneider’s medium only from absorbance readings of treatment plates. Significant differences between treatments are represented by horizontal bars above each column for each timepoint (t-tests, * *p* < 0.05, ** *p* < 0.005, *** *p* < 0.0005).

**Figure 3:**
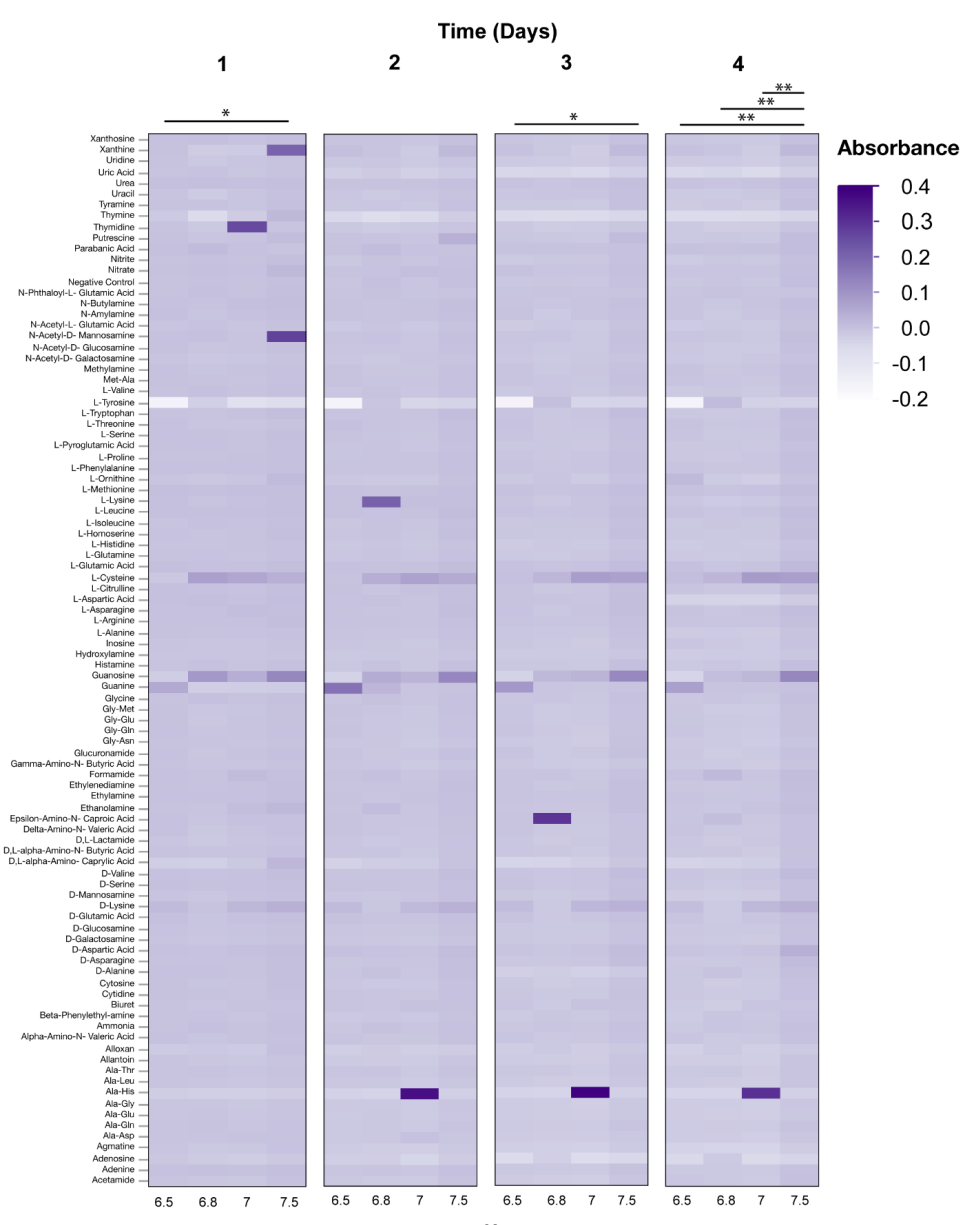
Heat map of PM3 phenotypic microarray data from *Wolbachia* isolated and maintained in Schneider’s medium supplemented with 10% fetal bovine serum (FBS) at different pH levels (*n* = 3 for each treatment) All data were normalized by subtracting absorbance values of control plates containing Schneider’s medium only from absorbance readings of treatment plates. Significant differences between treatments are represented by horizontal bars above each column for each timepoint (t-tests, * *p* < 0.05, ** *p* < 0.005, *** *p* < 0.0005).

**Figure 4:**
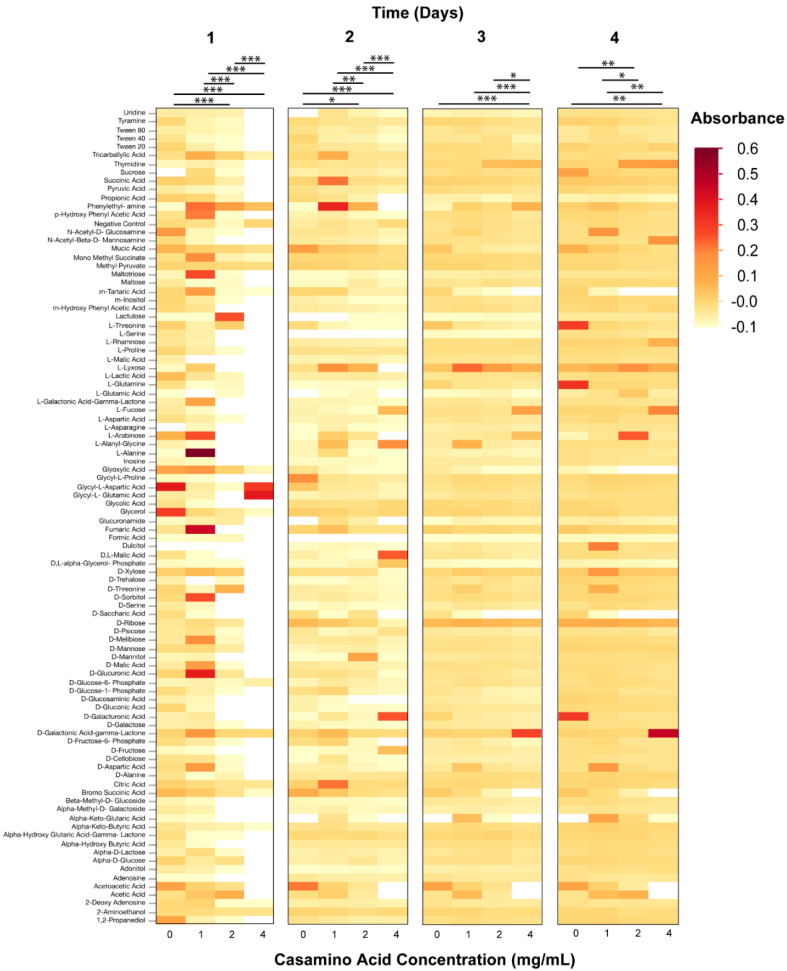
Heat map of PM1 phenotypic microarray data from *Wolbachia* isolated and maintained in Schneider’s medium supplemented with 10% FBS and different concentrations of Casamino acids (*n* = 3 for each treatment). All data were normalized by subtracting absorbance values of control plates containing Schneider’s medium only from absorbance readings of treatment plates. Significant differences between treatments are represented by horizontal bars above each column for each timepoint (t-tests, * *p* < 0.05, ** *p* < 0.005, *** *p* < 0.0005).

**Figure 5:**
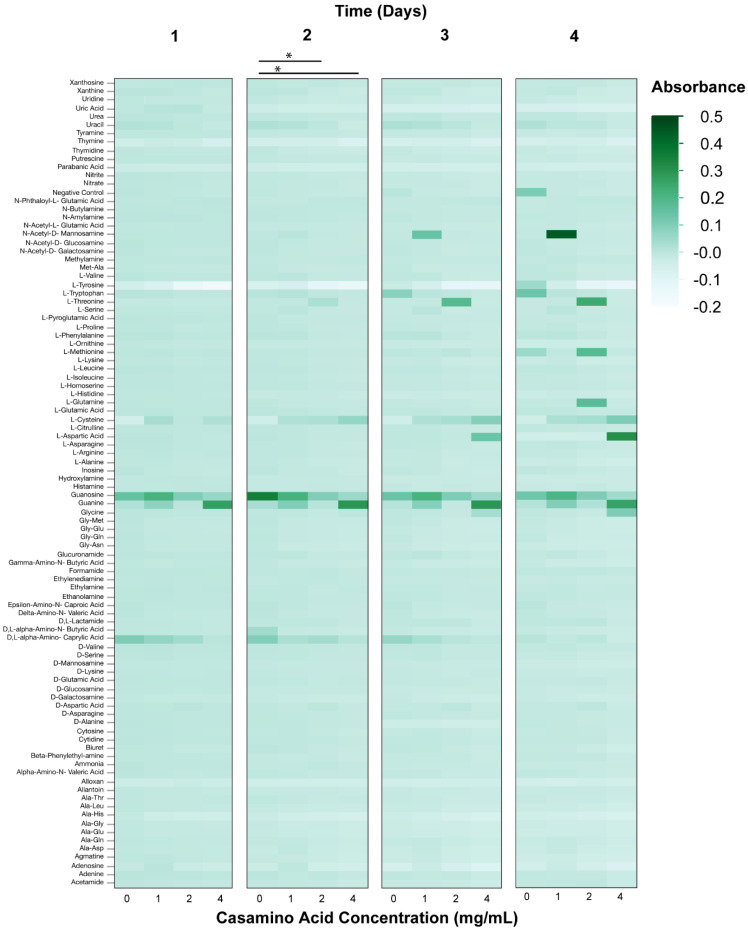
Heat map of PM3 phenotypic microarray data from *Wolbachia* isolated and maintained in Schneider’s medium supplemented with 10% FBS and different concentrations of Casamino acids (*n* = 3 for each treatment). All data were normalized by subtracting absorbance values of control plates containing Schneider’s medium only from absorbance readings of treatment plates. Significant differences between treatments are represented by horizontal bars above each column for each timepoint (t-tests, * *p* < 0.05, ** *p* < 0.005, *** *p* < 0.0005).

Phenotypic microarrays (PM) were used to measure the metabolic response of axenic *Wolbachia* to changes in pH over a four-day period. The range of normalized absorbance values (Au) of PM1 microarray plates were: pH 6.5—0.072–2.429, 6.8—0.073–1.32; 7.0—0.076–1.785; 7.5—0.065–1.779; Casamino acids 0 mg/mL—0.056–1.163, 1 mg/mL—0.061–1.221, 2 mg/mL—0.058–1.17, 4 mg/mL—0.054–1.992. The range of normalized absorbance values of PM3 microarray plates were: pH 6.5—0.051–0.451, 6.8—0.052–1.005, 7.0—0.05–1.402, 7.5—0.051–0.885; Casamino acids 0 mg/mL – 0.048–0.889, 1 mg/mL—0.05–1.477, 2 mg/mL—0.049–0.861, 4 mg/mL—0.051–1.168. Changes to media pH were demonstrated to impact the metabolic response of *Wolbachia* when added to PM1 plates (ANOVA, Day 1, F = 7.58, *p* < 0.0001; Day 2, F = 30.9, F < 0.0001; Day 3, F = 3.0, *p* = 0.0293; Day 4, F = 2.8, *p* = 0.0343). Media formulations of a pH of 6.5 and 6.8 typically had the highest metabolic response when examining absorbance values compared to media pH values of 7.0, and 7.5 (Figure 2). However, little differences in absorbance values were observed between the media formulations of different pH values when using PM3B plates (ANOVA, Day 1, F = 2.40, *p* = 0.0663; Day 2, F = 1.26, *p* = 0.2855; Day 3, F = 1.93, *p* = 0.1217; Day 4, F = 5.2135, *p* = 0.0014) (Figure 3). The metabolic response of axenic *Wolbachia* was also measured in response to changes in casamino acid concentrations in post-isolation media. Changes to media casamino acid concentration was demonstrated to impact the metabolic response of *Wolbachia* on PM1 plates (ANOVA, Day 1, F = 25.75, *p* < 0.0001; Day 2, F = 18.71, *p* = < 0.0001; Day 3, F = 7.05, *p* = 0.0001; Day 4, F = 5.10, *p* = 0.0016). When examining absorbance values, media formulations with a casamino acid concentration of 1 mg/mL had the highest metabolic response as compared to media formulations with no supplemented casamino acids, 2 mg/mL, and 4 mg/mL on PM1 plates (Figure 4). No difference in absorbance values were observed in media formulations with different casamino acid concentrations on PM3 plates (ANOVA, Day 1, F = 1.23, *p* = 0.30; Day 2, F = 2.39, *p* = 0.07; Day 3, F = 1.23, *p* = 0.30; Day 4, F = 0.66, *p* = 0.57) (Figure 5). 

For media formulations where pH or casamino acid concentration was altered, substrates on PM1 and PM3 plates associated with the TCA cycle and glycolysis were oxidized (i.e., Citric acid, Fructose-6-phosphate, Acetic acid), suggesting that these pathways are functional in extracellular *Wolbachia.* The presence of membrane transporters in *Wolbachia* genomes for proline, aspartate/glutamate, and alanine and pathways for metabolism of amino acids suggests that *Wolbachia* is dependent upon purine synthesis from amino acids. The oxidation of the amino acids (L-Alanine, L-Arabinose, L-Cysteine, L-Lyxose, L-Glutamine) and the purine guanine on PM3 plates support this hypothesis.

### 3.3. Quantitative Real-Time PCR

**Figure 6:**
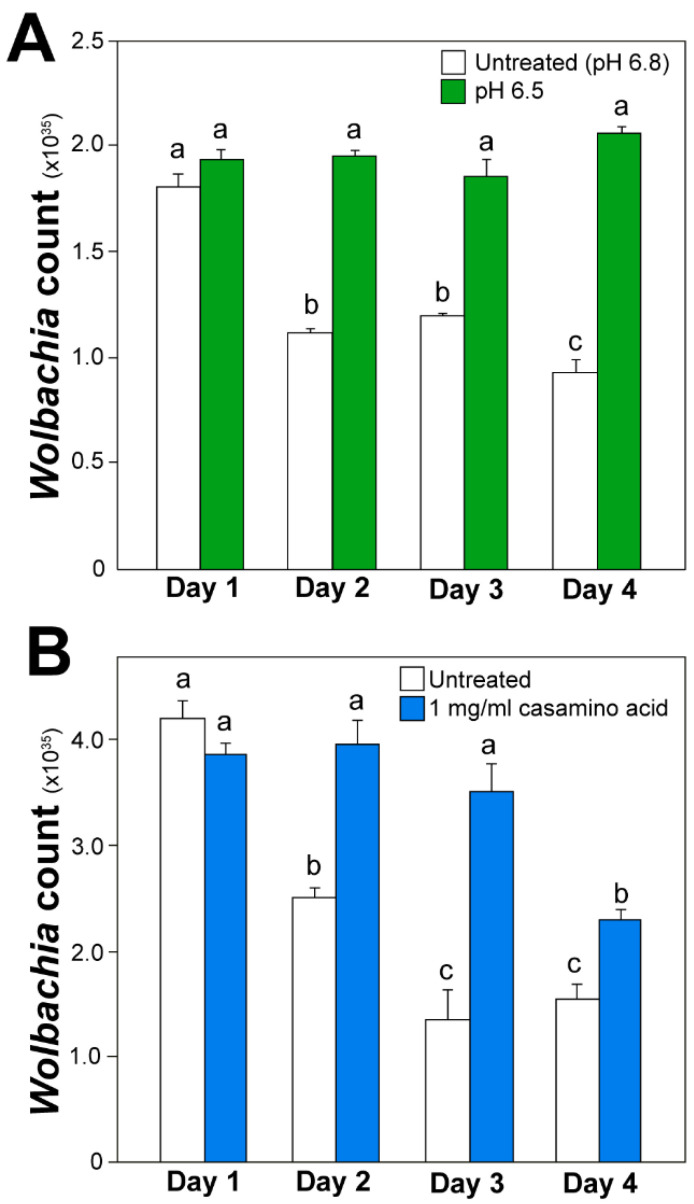
Extracellular *Wolbachia* count using quantitative polymerase chain reaction (qPCR) in (**A**) Schneider’s medium with 10% FBS adjusted to a pH of 6.5, and (**B**) Schneider’s medium with 10% FBS and 1 mg/mL Casamino acids. Significant differences are indicated by different letters above each bar (Mann–Whitney, *p* < 0.05).

Phenotypic microarrays suggest that a pH of 6.5 is optimal for an axenic medium. To investigate *Wolbachia* survival in an axenic medium with a pH of 6.5, qPCR was used to quantify the number of *Wolbachia* cells post-isolation over a four-day period. A greater number of *Wolbachia* copies were observed in an axenic medium with a pH of 6.5 compared to compared to unaltered Schneider’s media + 10% FBS (pH 6.8) (Kruskal–Wallis, Chi-sq = 9.0, *p* = 0.01). Results suggest an increase in the survivorship and longevity of extracellular *Wolbachia* in an axenic medium supplemented with a pH of 6.5 (Figure 6). qPCR was also used to quantify the number of *Wolbachia* cells over a four-day period in an axenic *Wolbachia* medium supplemented with 1 mg/mL of casamino acids, which showed the greatest metabolic activity in the phenotypic microarray assays. A greater number of *Wolbachia* copies were observed in an axenic medium supplemented with 1 mg/mL of casamino acids compared to unaltered Schneider’s media + 10% FBS (Kruskal-Wallis, Chi-sq = 8.4, *p* = 0.2). Results suggest an increase in the survivorship and longevity of extracellular *Wolbachia* in an axenic medium supplemented with 1 mg/mL of casamino acids (Figure 6). 

### 3.4. Wolbachia Cell Counts

**Figure 7:**
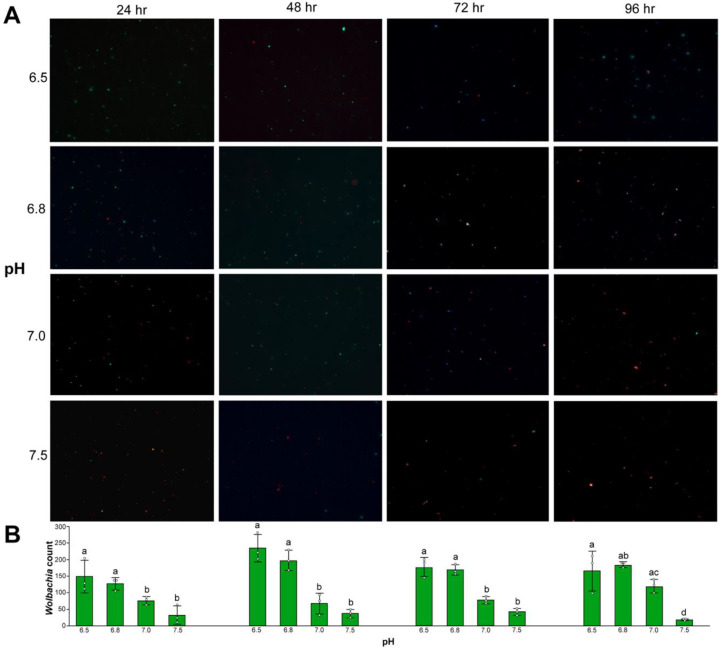
(**A**) Isolated *Wolbachia* stained with Baclight LIVE/DEAD™ viability assays in Schneider’s medium supplemented with 10% FBS and an adjusted pH of 6.5, 6.8, 7.0, and 7.5. Live and dead *Wolbachia* are green and red, respectively. (**B**) Live *Wolbachia* was counted using Image-J from three representative fields of view at 100x. Significant differences are indicated by different letters above each bar (Mann–Whitney, *p* < 0.05).

**Figure 8:**
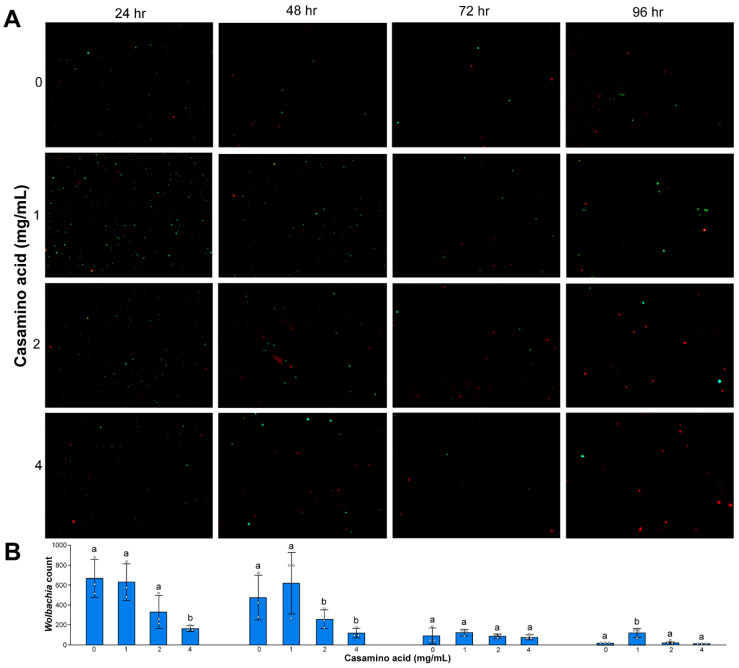
(**A**) Isolated *Wolbachia* stained with BacLight LIVE/DEAD™ viability assays in Schneider’s medium supplemented with 10% FBS and casamino acid concentrations of 0 mg/mL, 1 mg/mL, 2 mg/mL, and 4 mg/mL. Live and dead *Wolbachia* are green and red, respectively. (**B**) Live *Wolbachia* was counted using Image-J from three representative fields of view at 100x. Significant differences are indicated by different letters above each bar (Mann–Whitney, *p* < 0.05).

Baclight LIVE/DEAD™ images and cell counts were used as an additional metric to measure *Wolbachia* survivorship and longevity in axenic media formulations. Cell counts suggest a greater number of living *Wolbachia* cells in axenic media formulations with a pH of 6.5 and 6.8 at one, two, and three days post-*Wolbachia* isolation (Day 1, Kruskal–Wallis, Chi-sq = 8.94, *p* = 0.03; Day 2, Kruskal–Wallis, Chi-sq = 8.95, *p* = 0.03; Day 3, Kruskal–Wallis, Chi-sq = 9.46, *p* = 0.02; Day 4, Kruskal–Wallis, Chi-sq = 7.76, *p* = 0.05) (Figure 7). Cell counts also suggest a greater number of living *Wolbachia* cells in axenic media formulations when supplemented with 1 mg/mL of casamino acids (Day 1, Kruskal–Wallis, Chi-sq. = 8.23, *p* = 0.04; Day 2, Kruskal–Wallis, Chi-sq. = 8.3, *p* = 0.04; Day 3, Kruskal–Wallis, Chi-sq. = 2.3, *p* = 0.51; Day 4, Kruskal–Wallis, Chi-sq. = 9.4, *p* = 0.02) (Figure 8).

## 4. Discussion

Understanding the phenotypic response of *Wolbachia* outside of its host cell is an important first step in the development of an axenic *Wolbachia* culture and may help lead to the development of a *Wolbachia*-based paratransgenic interventions for insect vector borne diseases. *Wolbachia*-based paratransgenic approaches have remained theoretical because *Wolbachia* has yet to be genetically transformed inside or outside the host cell [48,49,50]. Separation of *Wolbachia* from its host cell in an axenic culture will facilitate the genetic transformation of *Wolbachia* by providing an axenic system to select for transformed *Wolbachia* cells. Selected transformed cells can reintroduced back into aposymbiotic cell lines for propagation. Furthermore, an axenic culture system will support additional studies to determine which *Wolbachia* genes and proteins are involved in host reproductive modifications, as well as potentially direct *Wolbachia* effects on pathogen disruption.

The observed metabolic activity of *Wolbachia* in the phenotypic microarrays provides a valuable tool to measure metabolic response to different media formulations and growth conditions outlined. The results from the phenotypic microarrays have identified several key amino acids and metabolites that require testing as supplements to axenic media formulations. Several of the compounds shown to have higher metabolism by *Wolbachia w*AlbB are a potential for precursors in the Kreb’s cycle, as well as glycolysis. Also, since *Wolbachia* lacks any ATPase machinery and is known to rely on glycolysis to generate adenosine monophosphate (AMP), guanosine monophosphate (GMP), and xanthosine monophosphate (XMP), this suggests that *Wolbachia* may derive energy from amino acids [18,21]. Purine ring containing compounds like ribose, lyxose, and xylose could be potential precursors of adenosine triphosphate (ATP), guanosine triphosphate (GTP), and cyclic-AMP that may be beneficial for the *Wolbachia* maintenance and survival [18,51,52,53,54,55,56,57]. In addition, from studies on *Wolbachia* genomes, it is known that there are multiple proline, aspartate/glutamate, and alanine membrane transporters present in *Wolbachia* [18,54,55,58,59]. The known *Wolbachia* pathways of amino acid metabolism suggest that *Wolbachia* is dependent on purine synthesis for the amino acids. The oxidation of the above-mentioned amino acids on the PM3B plates also supports this hypothesis. Overall, this suggests a reliance of *Wolbachia* on host amino acids and less reliance on carbohydrates as a major source of its energy. Previous genomic studies have drawn similarities between *Wolbachia* and *Rickettsia*, where both have limited carbohydrate metabolism, lipid biosynthesis, and amino acid transport capabilities [18,19,20,21].

Changes in pH in an axenic medium may also play an important role in *Wolbachia* survival outside of its host cell. An observed increase in *Wolbachia* longevity and survivorship and increased metabolic activity on phenotypic microarrays suggests that an axenic medium consisting of a pH of 6.5–6.8 may be most appropriate for *Wolbachia* survival outside of its host cell. The observed increase in *Wolbachia* longevity and survivorship, and increased metabolic activity on phenotypic microarrays, suggest a 1 mg/mL of casamino acids may also be an important component in an axenic medium formulation by providing amino acid precursors for energy production in addition to the components and amino acids in Schneider’s media (Table S1). Collectively, the data suggests that a medium consisting of Schneider’s insect media, 10%FBS, 1 mg/mL Casamino acids, and a pH of 6.5-6.8 is the best suited for maintaining viable extracellular up to four days. Additional experiments with the aforementioned medium formulation are required to determine if the medium formulation identified in this work will sustain *Wolbachia* longer than previous reports of seven days [13].

The identified medium conditions, as well as other metabolites identified on phenotypic microarrays, suggest the potential to develop an axenic medium to support the survivorship and potential replication of *Wolbachia* outside of its host cell. Whereas replication of *Wolbachia* in an axenic medium would be a significant research achievement and would open multiple avenues for future research, this may not be possible due to the intracellular nature of *Wolbachia* and reliance of *Wolbachia* on its host cell for its machinery and nutrients. However, if *Wolbachia* could be maintained outside of the host cell for a period of time and remain viable, *Wolbachia* could be transformed, and the transformed cells could be selected and reintroduced back into a novel insect or host cell line. Future work will focus on increasing the longevity and survivorship outside of the host cell with the ultimate goal of *Wolbachia* replication in an axenic medium. The observed results from this study also provide insight into the development of methodology to culture intracellular pathogens, such as closely related *Rickettsia* species, as well as *Chlamydiae* species, which result in Rocky Mountain spotted fever (RMSF) and chlamydial infections, respectively. 

## 5. Conclusions

In conclusion, we demonstrated that *Wolbachia* can be isolated and its metabolic activity can be measured using phenotypic microarrays. In addition, we demonstrated that amino acid supplementation, as well as adjusting pH similar to the internal environment of an insect cell, impacts the phenotypic response of *Wolbachia* and increases the survivorship of *Wolbachia* outside of its host cell. Future work will incorporate identified metabolites from phenotypic microarrays with different media formulations in an attempt to develop an axenic medium that will extend the survivorship and longevity of *Wolbachia* and/or examine for *Wolbachia* replication outside of the host cell.

## Supplementary Materials

The following are available online at https://www.mdpi.com/2076-2607/8/7/1060/s1, Table S1: Schneider’s media formulation (Sigma–S0146), Table S2: Primer sequences used to assay *Wolbachia* and other potential bacterial infections in cell cultures and *Wolbachia* extractions, Table S3: *Wolbachia* isolate bacterial infections status determined by PCR.
