# Supplementary material for: Phenotypic Response of Wolbachia pipientis in a Cell-Free Medium
Source: Microorganisms. 2020 Jul 16;8(7):1060. doi: 10.3390/microorganisms8071060 (PMC7409048; doi:10.3390/microorganisms8071060)
Supplement: Supplementary file 1 [file microorganisms-08-01060-s001.pdf]

Table S1. Insect Schneider's Media formulation (Sigma - S0146)

**Media components**	
**Inorganic Salts**	**g/L**
$CaCl_2$ (anhydrous)	0.6
$MgSO_4$ (anhydrous)	1.807221
KCl	1.6
$NaHCO_3$	0.4
NaCl	2.1
Na_2HPO_4	0.7
**Amino acids**	
β-Alanine	0.5
L-Arginine	0.6
L-Aspartic Acid	0.4
L-Cystine · 2HCl	0.026732
L-Cysteine	0.06
L-Glutamic Acid	0.8
L-Glutamine	1.8
Glycine	0.25
L-Histidine	0.4
L-Isoleucine	0.15
L-Leucine	0.15
L-Lysine	1.65
L-Methionine	0.15
L-Proline	1.7
L-Serine	0.25
L-Threonine	0.35
L-Tryptophan	0.1
L-Tyrosine · 2Na · $2H_2O$	0.7202
L-Valine	0.3
**Vitamins and misc.**	
Fumaric acid	0.06
D(+)-Glucose	2.0
α-Ketoglutaric acid	0.35
L-(-)-Malic acid	0.6
Succinic acid	0.06
D(+)-Trehalose	2.0
Yeast Extract	2.0

Table S2. Primer sequences used to assay *Wolbachia* and other potential bacterial infections in cell cultures and *Wolbachia* extractions.

Organism	Target gene	Primer sequence	Reference
Wolbachia	Wsp	**wsp-F:** 5′-ATCTTTTATAGCTGGTGGTGGT-3′<br>**wsp-R:** 5′- GGAGTGATAGGCATATCTTCAAT-3′	[60]
Wolbachia	*438 rDNA*	**Wspec-F**: 5′- CATACCTATTCGAAGGGATAG- 3′<br>**Wspec-R**: 5′- AGCTTCGAGTGAAACCAATTC- 3′	[61]
Acinetobacter	16s rDNA	**Acinetobacter-F:** 5′-ACTTTAAGCGAGGAGGAGGCT- 3′<br>**Acinetobacter-R:** 5′-GCGCCACTAAAGCCTCAAAGGCC- 3′	[62]
Asaia	16s rDNA	**Asaia-F:** 5′-AGCGTCAGTAATGAGCCAGGTT-3′<br>**Asaia-R**: 5′-GCGCGTAGGCGGTTTACA-3′	[63]
Cardinium	16s rDNA	**Car-F:** 5′-CGGCTTATTAAGTCAGTTGTGAAATCCTAG-3′<br>**Car-R:** 5′-TCCTTCCTCCCGCTTACACG-3′	[64]

Table S3. *Wolbachia* isolate bacterial infection status determined by PCR.

Organism	Samples tested (n)	Infection status*
Asaia spp.	3	-
Cardinium spp.	3	-
Acinetobacter spp.	3	-
Wolbachia (wsp)	6	+
Wolbachia (438)	6	+

*All samples tested had the same infection status
